# A meta-Ethnography on Parents’ Experiences of the Internet As a Source of Health Information

**DOI:** 10.1177/23333936241259246

**Published:** 2024-07-30

**Authors:** Thale Strand, Thomas Westergren

**Affiliations:** 1 University of Stavanger, Norway; 2 University of Agder, Kristiansand, Norway

**Keywords:** health information seeking, internet, parenting, child health, parental health literacy, consumer health information

## Abstract

The Internet is increasingly being used as a health information resource. This meta-ethnography aimed to synthesize the literature on how parents of children aged below 5 years’ experienced using the Internet for health information purposes. We employed an interpretive meta-synthesis approach—meta-ethnography—according to Noblit & Hare’s seven phases. A total of 22 articles met the inclusion criteria, representing four continents and with 650 participants, mainly mothers. We analysed and synthesized the primary studies into the following lines-of-argument synthesis representing a novel conceptual understanding of the phenomenon: Parents experience the Internet as “A cyber partner for child caring” being a 24/7 available “go-to” among other confined sources. Parents find ways of “patching together” trustworthy information in solicitude for their child’s health while navigating between trust and anxiety. They relate online and share their experiences and secrets without being rejected. Clinicians and parents may benefit from “partnering” with this resource.

## Introduction

The Internet may represent a lay referral system for parents to evaluate and complement parenting information from families, friends, and healthcare personnel (Loignon et al., 2022), and to navigate between lived experience and expert knowledge (Tian & Zhang, 2022). According to Walker (2012), parenting norms have consequences for how parents search for, access, and evaluate information. A recent review of scientific literature from the last two decades points on the existence of five main contemporary parenting norms: Being attentive and present; securing the child’s development and future; combining parenting and employment; being in control; and being contented/happy (Schmidt et al., 2023). The “information literacy” hence appears within a complex and socially constructed world of parenting, and Walker (2012) found in a qualitative study that connectivity to others, trust (in people and information), weighing (and reflection) of information, and picture (and picturing) of oneself reciprocally interact. A recent qualitative study by Tschamper et al. (2023) on how parents handle health information concerning their child points in a similar direction. Health literacy development is a continuous process; emotionally, cognitively, and socially. Throughout this paper, health information is defined as information about health, illness, and healthcare (Mårtensson & Hensing, 2012). Health literacy represents the ability to access, understand, appraise, and use information and services to promote and maintain their own and others’ health and well-being (WHO, 2021).

A literature review revealed two different approaches in the literature about health literacy: as a polarized phenomenon and as a complex phenomenon (Mårtensson & Hensing, 2012). Health literacy as a polarized phenomenon was assessed as high and low levels of skills in reading, writing, and numeracy to understand health information and the functional ability within the healthcare environment, often linked directly to high or low ratings of health (Mårtensson & Hensing, 2012). Health literacy as a complex phenomenon represented a more dynamic approach to the concept, and levels may fluctuate depending on the cultural and social context. Health literacy not only includes functional skills related to the healthcare system as a basis for maintaining good health and making appropriate health decisions, but also interactive and critical skills, and in all contexts of human life (Mårtensson & Hensing, 2012).

In this paper, health information and literacy relate to the context of contemporary parenting, briefly described above, as well as the Internet with its’ wealth of information of various quality (Chu et al., 2017). Worthy of notion, Facebook was founded in 2004 and many social media platforms has followed (Enli & Aalen, 2021), and Google, hosting the world’s most used search engine, was listed in the same year (Abrahamsen & Gramstad, 2019). At present, 60% of the world’s population has access to the Internet, ranging from 30% in Sub-Saharan Africa to 90% in North America closely followed by Europe and Central Asia (Ritchie, 2023).

In a recent qualitative study on maternal experiences with online information on parenting and childcare, Loignon et al. (2022) reported that parents strategically use the Internet for better parenting, have a critical stance toward different sources, and that the Internet may strengthen parental autonomy, skills, and self-confidence. Findings from previous research also suggest that the Internet serves as a supplement to healthcare services, rather than as a replacement (Yigzaw et al., 2020). During the recent Covid-19 pandemic, parental stress and worry increased (Spinelli et al., 2020), and health information searching increased substantially (Bento et al., 2020). Negrone et al. (2023) reported that, during the pandemic, parents had a dynamic relationship with online resources which was the preferred information source.

Three previous reviews concerning parental experiences and practices of searching the Internet for health information concerning their children have recently been published (Kubb & Foran, 2020; Pretorius et al., 2019; Frey et al., 2022). The review of Kubb and Foran (2020) included quantitative studies and reported that 52 to 98% of parents used online resources for health information mostly using Google to understand their child’s condition and decide whether to contact health services. The less disease specific focus, the greater variety in search content were reported. Searching for support groups was common. Parents reported that health information on the Internet was easy to understand, whereas there was a variation between studies in how and whether reliability and trustworthiness were considered by parents. Moreover, parents did not necessarily navigate to the most trusted websites and did not discuss their findings with physicians despite wishing for more guidance. Even though some parents reduced their anxiety levels by online searching, increased fear was twice more common. Authors finally reported that there is a lack of understanding about parents’ reasoning when making decisions based on what they find on the Internet, and how information on the Internet may contribute to empower parents (Kubb & Foran, 2020).

The integrative review of Pretorius et al. (2019) focused on parental use of social media, reporting Facebook as the most common format and infant feeding practices as the most common topic, with variations related to race/ethnicity and study region (Pretorius et al., 2019). Facebook and YouTube were, by mothers, considered supportive and an effective way to obtain parenting and health information. Authors, however, requested more in-depth research to further understand variations between ethnic groups and contexts in utilization of social media for parenting and health information (Pretorius et al., 2019).

Lastly, a scoping review by Frey et al. (2022), investigated parental use of social media for health information purposes, their motivation for use, and how parents understand and seek further information. Parents sought information on social media to address health concerns before and after a medical diagnosis and were motivated by gaining access to lived experience from other parents, social support, and community. Social media platforms were reported to provide immediate and detailed knowledge, and a safe place to discuss sensitive topics. Contrarily, social media created discomfort and conflict, and provided unhelpful information concerning worst-case scenarios, and misleading information. Parents also reported quality concerns which they could address by gaining information directly from peer-reviewed journals, triangulation through crowdsourcing, and verification by professionals. Authors conclude that parents use social media to a great extent, and that there is a need to combat misinformation by giving parents appropriate training (Frey et al., 2022).

Although the abovementioned reviews recently have provided insights into, and overview of, the published quantitative and qualitative literature on parental use of the Internet for health information purposes, we have found no interpretive qualitative synthesis examining this matter across different study populations and contexts throughout the last two decades of parenting that reflects contemporary parenting norms and internet use. Therefore, to gain an in-depth insight into parental experiences of the Internet as a health information resource, the aim of this meta-ethnography was to synthesize the literature on how parents of children aged below 5 years experienced using the Internet for health information purposes.

## Methods

### Design

We conducted this meta-synthesis as a meta-ethnography, which is an approach that was developed by Noblit and Hare (1988), consisting of seven phases. We used the eMERGe reporting guidelines for meta-ethnography developed by France et al. (2019) for clarifications and additional information about how to implement and transparently report the method within the seven phases and a total of 19 recommended reporting criteria. Additionally, we utilized the meta-ethnography by Britten et al. (2002) as a reference paper for how to conduct and report the current study. We will list the seven phases and our applications of these in the study in the next section, but it is important to note that, in the execution of this process, the phases overlapped and did not follow a linear process (Noblit & Hare, 1988, p. 29).

### Data Collection and Analysis

Phase 1: Getting started. When initiating this project, our preconceptions were that parents frequently use the Internet to obtain health information, and that they find it difficult to evaluate the trustworthiness of the information they find. We developed the aim of this study over time during the process of reading research reports about the topic.

Phase 2: Deciding what is relevant. During the initial process of finding relevant studies, we developed the inclusion and exclusion criteria. We included research exploring the use of the Internet as a health information resource from a parental perspective. Studies exploring this topic from the perspective of children, youths or healthcare professionals were excluded. We included peer-reviewed qualitative studies (Noblit & Hare, 1988) of parents who had children aged below 5 years and that had been published in English or a Scandinavian language. Studies including pregnant women or parents of children aged above 5 years were not excluded, as long as they also included experiences of parents of children aged below 5 years that were possible to isolate and extract from the findings. To cover the last two decades of parenting and internet use, we included studies from 2004 to 2023.

**Figure 1.**
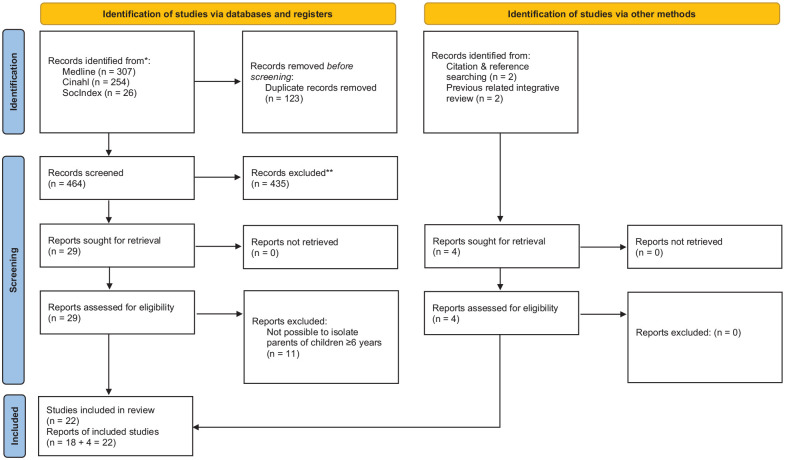
PRISMA flow diagram of records identified, screened for eligibility, and included in the meta-ethnography (Page et al., 2021).

We consulted a research librarian in planning the database searches, and conducted database searches in CINAHL, Medline, and SocIndex (EBSCO host interface) by using the terms (parent* OR mother* OR father*) AND (“social media” OR internet OR “mobile application*” OR “online behaviour*” OR “digital media” OR “discussion forum*” OR Facebook OR Instagram OR TikTok OR Snapchat OR Telegram) AND (“health information” OR “information seeking behaviour*” OR “health litera*”) AND (qualitative OR interview* OR “focus group*” OR experience* OR belie* OR naturalistic). We also screened reference lists and conducted a forward citation search (in Google Scholar) of identified and included articles. This process is depicted in the flow chart (PRISMA) (Page et al., 2021) in Figure 1. We completed the last updated search on May 9, 2023. Excluded full-text articles with reasons are given in Supplemental File 1. Both authors screened titles and abstracts for eligibility using Rayyan, and individually assessed full texts for inclusion.

Phase 3: Reading the included studies in full text. In this phase, we carefully read the studies several times (Noblit & Hare, 1988, p. 28). We identified and recorded concepts from the primary studies using the NVivo software (QSR International, Burlington, MA, USA). By concepts, we mean the main findings and concepts that were reported in the included studies and that were relevant to the research aim.

We only included studies that were published in peer-reviewed journals in this meta-synthesis and did not exclude studies based on critical appraisal. Each author critically evaluated the studies using the well-known “critical appraisal skills programme” (CASP) tool for qualitative research articles (CASP, 2018). Item 10 stated as “How valuable is the research?” was not included in the table as it cannot be answered with a “yes” or “no.” Discrepancies between authors’ evaluation were resolved by discussion.

We reviewed the ethical assessments in each of the primary studies included in the current meta-ethnography (Støren, 2013, p. 34). During the process of writing this paper, we have been aware of our ethical responsibility, although no ethics approval was required for this meta-ethnography. By trying to remain faithful to the primary studies, and to avoid the creation of misunderstanding or distortion in the current meta-synthesis, we have aimed to retain the subjective perspective from the primary studies on which the synthesis relies (France et al., 2019).

Phase 4: Determine the relationship between the studies. Early in this process, we recorded details about the study setting, method, participants, and theory in different tables in order to provide the context for the interpretations and explanations of each study (Britten et al., 2002; France et al., 2019) (see Table 1 and Supplemental File 2, Table 1x).

**Table 1.** Summary of Characteristics of the 22 Included Studies.

Study origin	Europe, North America, Asia, Oceania (21 out of 22 in Western countries)
Study year (range)	2004−2023
Study participants (*n* = 650)	Share in each study (*n*): 10−153 Mothers (*n* = 615) Fathers (*n* = 34) Sister (*n* = 1)
Educational level of participants	Share of participants with a university/college degree: 12−89% Studies with a majority of participants holding a university/college degree (*n* = 9 (range 60−89% to “most”)) Studies not reporting educational level (*n* = 6)
Age of participants (range)	18−57 years Studies not reporting age (*n* = 5)
Data collection methods utilized	Individual interviews (*n* = 16) Focus groups (*n* = 9) Open-ended questionnaire (*n* = 1)
Data analysis methods utilized	Thematic analysis (*n* = 7) Content analysis (*n* = 4) Grounded theory (*n* = 2) Constant comparison (*n* = 3) Inductive cut and paste with deductive matrix (*n* = 1) Interpretive phenomenology (*n* = 1) Phenomenographic analysis (*n* = 1) Discourse analysis (*n* = 1) Immersion/crystallization (*n* = 1) No analysis method reported (*n* = 2)
Theoretical approaches	Information seeking (*n* = 3) Health education and support (*n* = 3) Decision making (*n* = 2) Feminism (n = 3) Empowerment (*n* = 1) Diffusion of innovations theory (*n* = 1) Intervention (*n* = 1) Theory of planned behavior (*n* = 2) Digital health literacy (*n* = 1) Social constructionism (*n* = 1) Socioeconomic status (*n* = 2) Race/ethnicity (*n* = 1) Sociocultural and political aspects (*n* = 1) “Weak ties” (*n* = 2) “Intra-action” (*n* = 1) Virtual publics and counter publics (*n* = 1) Health anxiety (*n* = 1)

We carefully read studies and used NVivo to code the studies, and initially formulated sentences that could summarize the key concepts in every study, using the primary studies’ own terminology. Instead of naming each line of written data, we performed line-by-line coding (Charmaz, 2014, p. 50), naming all parts of data (in the form of a sentence) that revolved around the similar phenomenon. We applied this procedure to all 22 of the included studies. At the end of this phase, we made an assumption about the reciprocal and/or refutational relationship between the studies (Noblit & Hare, 1988, p. 36).

By comparing those sentences describing findings from each study, we developed common and recurring concepts at the end of this phase (Britten et al., 2002). We then created several tables (Supplemental File 3, Table 4–6) with these key concepts. When developing the tables, we used Schutz’s notion of first, second and third order interpretations (Atkins et al., 2008; Britten et al., 2002). First order interpretations reflect the understanding of the participants of the original studies, second order interpretations reflect the authors’ interpretations of the participants’ understanding in the original studies, and third order interpretations reflect the synthesis of first and second order interpretations to construct a new model or theory about the phenomenon (Atkins et al., 2008). Supplemental File 3 (Tables 4−6) presents the first order interpretations, while Table 3 in this document presents the second and third order interpretations.

Phase 5: Translating the studies into one another. In this phase, we connected all the studies by including them in several tables, with a separate row for each paper (Supplemental File 2, Table 1x and Supplemental File 3, Table 4─6) (Noblit & Hare, 1988, p. 28). The empty cells in the table represent the absence of information about the particular concept in the paper concerned (Britten et al., 2002). We paraphrased the second order interpretations that had been formulated in NVivo to form shorter sentences or terminology that still included the meaning of the concepts used in the original studies, as a way of remaining faithful to them (Britten et al., 2002). Each cell of the grid was considered in turn. We started by identifying the actual concepts described in the original papers, and then made sure that the concepts encompassed with the key concepts that were developed to label that row of the table (Britten et al., 2002). Sometimes, we borrowed the key concept terminology from one of the original papers (for instance, the term “shared experiences” was borrowed from Madge & O’Connor, 2006, and the term “go-to” was borrowed from a participant’s quote in Henshaw et al., 2018). In this phase, we carefully discussed and evaluated study differences in study characteristics, methods, and study quality, as well as our preconceptions to reduce the risk of unintentionally reproducing any bias in our interpretations. Both reviewers are trained nurses by profession. The first author is a novel researcher and a mother of two children born the recent 3 years and have firsthand (positive and negative) experiences of contemporary use of the Internet for parenting information. The second author is an experienced health and nursing science researcher and a father of three children born two decades ago, being an observer of parental use of the Internet for health information purposes both privately and professionally.

Phase 6: Synthesizing translations. By reading the concepts and interpretations of Table 3 and Supplemental File 3 (Tables 4−6), we established the relationship between the 22 included studies (Britten et al., 2002), and a lines-of-argument synthesis was developed to gain a novel conceptual and clinically relevant understanding of the whole (Noblit & Hare, 1988).

Phase 7: Expressing the synthesis. This paper is an attempt to express the synthesis in a way that is as easy as possible for the reader to understand. We have also tried to remain faithful to the original data throughout the process of writing this paper (Britten et al., 2002).

## Results

### Characteristics of the Included Studies

A summary of characteristics across the included studies are presented in Table 1. The 22 articles included represented four continents and were published during the period between 2004 and 2023. Notably, 21 out of 22 studies were conducted in Western countries. The total numbers of participants were 650 (615 mothers), with one study (Madge and O’Connor, 2006) that did not report the numbers of mothers participating. In nine studies, a majority of the participants had a university or college degree. Data collection was conducted using individual (16 studies) or focus group interviews (9 studies), and one study had virtual group interviews (Madge & O’Connor, 2006), one conducted online electronic interviews (Aston et al., 2018), and one were based on an open-ended questionnaire (Rathbone & Prescott, 2019). Data analysis applied varied between inductive and deductive approaches, as well as between interpretive and merely descriptive approaches. Detailed study characteristics are presented in Supplemental File 2, Table 1x. We made an assumption that a combination of individual and lived experiences as well as the social interaction and negotiation of the phenomenon studied could be well supported by the mix of individual and group-based data collection methods across the studies. Similarly, we assumed that variations in data analysis methods across the 22 studies comprehensively could support our synthesis, providing us with rich data from the descriptive level of experiences, as well as the more interpretive, and from inductive and deductive approaches. In the analysis of the studies, we made an assumption about the relationship between the studies being reciprocal.

### Critical Appraisal of the Included Studies

The evaluation of the quality of the studies are presented in Table 2. Studies included were evaluated with satisfactory quality in most aspects. However, 13 out of 22 studies did not report adequately about the relationship between the researcher and the participants, in which author bias may have been unintentionally introduced in analysis and findings. Despite the different methodology approaches in these 22 studies and flaws identified by our quality assessment in 13 studies, the results pointed in the same directions including the nine studies with good credibility.

**Table 2.** Critical Appraisal Skill Program Assessment of Included Qualitative Studies.

Author(s), (year)	Item 1	Item 2	Item 3	Item 4	Item 5	Item 6	Item 7	Item 8	Item 9
Alianmoghaddam et al. (2019)	Yes	Yes	Yes	Yes	Yes	Yes	Yes	Yes	Yes
Altawil et al. (2023)	Yes	Yes	Yes	Yes	Yes	Yes	Yes	Yes	Yes
Aston et al. (2018)	Yes	Yes	Yes	Yes	Yes	Yes	Yes	Yes	Yes
Bäckström et al. (2021)	Yes	Yes	Yes	Yes	Yes	No	Yes	Yes	Yes
Bernhardt and Felter (2004)	Yes	Yes	Yes	Yes	Yes	No	Yes	Yes	Yes
Casilang et al. (2020)	Yes	Yes	Yes	Yes	Yes	No	Yes	Yes	Yes
Clapton-Caputo et al. (2021)	Yes	Yes	Yes	Yes	Yes	Yes	Yes	Yes	Yes
Criss et al. (2015)	Yes	Yes	Yes	Yes	Yes	No	Yes	Yes	Yes
Griauzde et al., (2020)	Yes	Yes	Yes	Yes	Yes	No	Yes	Yes	Yes
Guerra-Reyes et al. (2016)	Yes	Yes	Yes	Yes	Yes	No	Yes	Yes	Yes
van der Gugten et al. (2016)	Yes	Yes	Yes	Yes	Yes	No	Yes	Yes	Yes
Henshaw et al. (2018)	Yes	Yes	Yes	Yes	Yes	Yes	Yes	Yes	Yes
Johnson (2015)	Yes	Yes	Yes	Yes	Yes	No	Yes	Can’t tell	Yes
Lupton (2016)	Yes	Yes	Yes	Yes	Yes	Yes	Yes	Yes	Yes
Madge and O’Connor (2006)	Yes	Yes	Yes	Yes	Yes	Yes	Yes	Can’t tell	Yes
Maslen and Harris (2021)	Yes	Yes	Yes	Yes	Yes	Yes	Can’t tell	Yes	Yes
Moon et al. (2019)	Yes	Yes	Yes	Yes	Yes	No	Yes	Yes	Yes
Neill et al. (2014)	Yes	Yes	Yes	Yes	Yes	Yes	Yes	Yes	Yes
Rathbone and Prescott (2019)	Yes	Yes	Yes	Can’t tell	Yes	No	Yes	Can’t tell	Yes
Sharma et al. (2022)	Yes	Yes	Yes	Yes	Yes	No	Yes	Yes	Yes
Sundstrom (2016)	Yes	Yes	Yes	Yes	Yes	No	Yes	Yes	Yes
Wagg et al. (2022)	Yes	Yes	Yes	Yes	Yes	No	Yes	Yes	Yes

*Note.* Items: 1. Was there a clear statement of the aims of the research? 2. Is a qualitative methodology appropriate? 3. Was the research design appropriate to address the aims of the research? 4. Was the recruitment strategy appropriate to the aims of the research? 5. Was the data collected in a way that addressed the research issue? 6. Has the relationship between researcher and participants been adequately considered? 7. Have ethical issues been taken into consideration? 8. Was the data analysis sufficiently rigorous? 9. Is there a clear statement of findings?

### Synthesis and Interpretation

We constructed three third order interpretations from the translation and synthesis of first (see Supplemental File 3, Tables 4–6) and second order interpretations (see Table 3). These key concepts were: (1) The 24/7 available “go-to” among other confined sources, (2) “patching together” trustworthy information with solicitude, and (3) relating online through shared experiences (see Table 3). Those were grounded with the primary study data to avoid the loss of conceptual richness (France et al., 2019). In the following section, we will explain the meaning of the three main concepts that emerged during the translation process, and that have underpinned our third order interpretations and the lines-of-argument synthesis.

**Table 3.** Illustrations of Second Order Interpretations and Our Three Third Order Interpretations. Each Row Relates to Concepts Given in Detail in Supplemental file Tables 4 to 6.

Illustrations of second order interpretations in included primary studies	Third order interpretations
Parents appeared to be in need of information when their infant displayed symptoms, and because of its easy accessibility internet was a major source of information. (van der Gugten et al., 2016) Digital media provided women with details when they most needed them or at times when they had opportunities to access them. (Lupton, 2016) Digital sensory work, and in particular the learning involved by parents as they become diagnostic agents, does not take place separate from the clinic (Maslen & Harris, 2021) (Healthcare professionals should assume an active role in developing digital parental support, both as health educators and facilitators, and as a complement to standard care) (Bäckström et al., 2021) (Parents can access a wealth of information independently, but there is a need for clearly signposted, and professionally validated resources) (Neill et al., 2014)	#1: **The 24/7 available “go-to” among other confined sources**. The possibilites of the Internet was a source of empowerment for parents caring for their children and their health, being primarily like a “partner” and not a professional concerning availability, charging, and adjustability to parents’ needs. The Internet was always there for parents. Hence, the Internet was the 24/7 available “go-to” among other more confined sources for health information, such as relatives, friends, and healthcare providers.
The spread of pandemic-related information, (. . .) through a multitude of channels allowed parents to access and select sources that satisfy their information needs (Altawil et al., 2023) (Mothers critically analysed information and support to ensure it matched their own beliefs, values, and practices in mothering. When they were confident in their own maternal knowledge, they were able to challenge dominant social and institutional discourses regarding mothering practices, to do what felt best for them.) (Aston et al., 2018) (Internet was the primary source for information on infant care and provide knowledge, reassurance, and help to normalize a stressful transition) (Guerra-Reyes et al., 2016) Participants found information seeking to be a significant challenge that often contributed to feeling overwhelmed and unsure. (Henshaw et al., 2018) (New parents strive to expand their own knowledge base for better parenting. The online platform was used to compensate inexperience, avoid judgment, and allow anonymity.) Online health information seeking behavior has the probability of both increasing and decreasing levels of anxiety. (Rathbone & Prescott, 2019)	#2: **“Patching together” trustworthy information with solicitude**. The independent and critical navigation of parents concerning health information on the Internet made it a supportive “partner” for preferred advices they found trustworthy, and a “partner” companioning them in solicitude for their child’s health while navigating between trust and anxiety.
Participants both expected and experienced emotional support, and also received information and practical support while using a social media support group for exclusively expressing breastmilk to feed infant/s (Clapton-Caputo et al., 2021) (Mothers gather experiential information and practical support through “intimate mothering publics” and it can act as a space for women to “test” or legitimize their new identity as a mother.) (Johnson, 2015) Internet played a central role in providing virtual social support and alternative information sources which increased these women’s real sense of empowerment in the transition to motherhood (Madge & O’Connor, 2006) (Online groups offered the women emotional, technical, informational, and experiential support that provided the mothers with reassurance, normalization of breastfeeding and helped grow their confidence as a mother) (Wagg et al., 2022)	#3: **Relating online through shared experiences**. The Internet, with its’ cyber connections, represented a community of reciprocity providing tools for respectful communication and partnership with people who shared their secrets and never rejected their knowledge or experiences.

*Note.* Entries in parentheses are paraphrased to make shorter sentences, all other entries are the original author’s own words.

Although we identified studies as reciprocal, in one matter, however, two studies (Griauzde et al., 2020, Henshaw et al., 2018) were somewhat refutational to the other 20 studies, focusing more on the negative and challenging experiences of Internet use among parents than most studies. One of those studies addressed samples and theory focusing on a more disadvantaged population (Griauzde et al., 2020) and the other (although highly educated participants) on supporting needs (Henshaw et al., 2018). That contrasts with most studies reporting the experiences of parents, mainly mothers, who reported more positive perspectives about information seeking, decision making, and empowerment. On the interpretive and translational level of the synthesis, however, those refutations did not change the interpretations of experiences beyond variations of positive or negative experiences also within studies. Rather, they deepened the understanding of experiences across studies and study populations, reciprocally supporting our third order interpretations and the final lines-of-argument synthesis.

### The 24/7 Available “go-to” Among Other Confined Sources

The first concept, the 24/7 available “go-to” among other confined sources for health information, includes both the aspect of availability and convenience, as well as parental preferences for the internet as the place to “go-to.” Even though parents, mainly represented by mothers, trusted healthcare professionals (Alianmoghaddam et al., 2019; Altawil et al., 2023; Bernhardt & Felter, 2004; Casilang et al., 2020; Criss et al., 2015; Sharma et al., 2022; van der Gugten et al., 2016), the Internet constituted a practical and convenient source for information which was both affordable and accessible day and night throughout the week (Alianmoghaddam et al., 2019). The Internet was considered helpful, accurate, detailed, easy accesible, and sufficient (Altawil et al., 2023; Bäckström et al., 2021; Guerra-Reyes et al., 2016; Lupton et al., 2016; Madge & O’Connor, 2006; Wagg et al., 2022). A key aspect was that the Internet was used among, and as a supplemet to, other sources (Altawil et al., 2023; Aston et al., 2018; Bernhardt & Felter 2004; Clapton-Caputo et al., 2021; Criss et al., 2015; Johnson, 2015; Lupton, 2016; Maslen & Harris, 2021; Moon et al., 2019; Neill et al., 2014; Sharma et al. 2022; van der Gugten et al., 2016). Parents used Internet to prepare for (Bernhardt & Felter, 2004), evaluate, and follow-up encounters with healthcare professionals (Aston et al., 2018; Bernhardt & Felter, 2004; Casilang et al., 2020; Criss et al., 2015; Johnson, 2015; Madge & O’Connor, 2006; Moon et al., 2019; Neill et al., 2014; van der Gugten et al., 2016). Specifically, the 24 hr availability was expressed (Clapton-Caputo et al., 2021; van der Gugten et al., 2016), or described as availability without closing or when healthcare professionals were unavailable (Alianmoghaddam et al., 2019; Criss et al., 2015; Madge & O’Connor, 2006). The Internet could also be utilized without bothering or engaging others (Lupton, 2016; Moon et al., 2019) and was used for at plethora of topics (Rathbone & Prescott, 2019). Information on the Internet was experienced as reflecting a “layperson” expertise (Sundstrom, 2016), was helpful in decision making (Casilang et al., 2020), free of charge (Bäckström et al., 2021), and included several types of sources, such as social media, applications (Griauzde et al., 2020), forums (Moon et al., 2019), online searching (Rathbone & Prescott, 2019), and communication channels (Sundstrom, 2016).

The supplement and layperson perspective of the Internet were also visible in parental preferences. Parents desired approved, signposted, and profesionally validated websites (Criss et al., 2015; Neill et al., 2014; Sundstrom 2016), and some parents wanted healthcare professionals to engage online (Bäckström et al, 2021), which according to Bernhardt & Felter (2004) represents an essential opportunity to reach parents with information. Parents preferred tailored information (Bernhardt & Felter, 2004; Henshaw et al., 2018; Moon et al., 2019; van der Gugten et al., 2016) and a possibility for a direct interaction with healthcare professionals online to get reliable answers immediately (Altawil et al., 2023; Bäckström et al., 2021; Casilang et al., 2020; Henshaw et al., 2018; Lupton, 2016). Overall, the possibilites of the Internet was a source of empowerment for parents caring for their children and their health, being primarily like a “partner” and not a professional concerning availability, charging, and adjustability to parents’ needs.

### “Patching Together” Trustworthy Information with Solicitude

The second concept, “patching together” trustworthy information with solicitude constitutes both the perspective of handling and evaluating the information for the health of their child, “patching” parts and perspectives together, as well as carefully dealing with the anxiousness and doubt. Parents, represented mainly by mothers, could personalize (Madge & O’Connor, 2006) and adjust information to their beliefs, values, and practices (Aston et al., 2018). They could “patch together” their own version (Johnson, 2015), and use the Internet for crowdsourcing information (Moon et al., 2019). Parents experienced that they used credible sources for health information (Casilang et al., 2020; Clapton-Caputo et al., 2021) and stated that they evaluated all health information sources for credibility and trustworthiness (Aston et al., 2018; Neill et al., 2014; Sundstrom, 2016). For parents, health information on the Internet was contributing with learning (Alianmoghaddam et al., 2019; Bernhardt & Felter, 2004; van der Gugten et al., 2016), reassurance, and validation (Casilang et al., 2020; van der Gugten et al., 2016; Wagg et al., 2022). Online informaton improved self-confidence and sense of control (Alianmoghaddam et al., 2019; Madge & O’Connor, 2006; Moon et al., 2019; Sharma et al., 2022). Information was used to oppose professionals when needed (Wagg et al., 2022), getting multiple viewpoints (Moon et al., 2019), developing a frame of reference (van der Gugten et al., 2016), resolving contradictory information (Criss et al., 2015), and making informed decisions (Aston et al., 2018; Bernhardt & Felter, 2004; Casilang et al., 2020; Johnson, 2015). Hence, they patched information together in ways that were trustworthy to their own experiences and needs.

In contrast, parents also found it challenging to find reliable information they could trust (Altawil et al., 2023; Bernhardt & Felter, 2004; Henshaw et al., 2018; van der Gugten et al., 2016), and were skeptical about information on the Internet (Alianmoghaddam et al., 2019; Sharma et al., 2022). Information could be conflicting leading to uncertainty and anxiety (Neill et al., 2014), and experienced as overwhelming and confusing (Henshaw et al., 2018; Johnson, 2015). Hence, parents were selective (Aston et al., 2018), avoided certain sources, for instance social media (Griauzde et al., 2020), apps with repetitive non-validated content (Guerra-Reyes et al., 2016), information lacking credibility and autheticity (Sharma et al., 2022), or too much information (Henshaw et al., 2018). The independent and critical navigation of parents concerning health information on the Internet made it a supportive “partner” for preferred advice they found trustworthy, and a “partner” companioning them in solicitude for their child’s health while navigating between trust and anxiety.

### Relating Online Through Shared Experiences

The third concept, relating online through shared experiences, constitutes the experience of the Internet as a platform for social interaction with other parents concerning health information. Parents, mainly represented by mothers, related to other likeminded parents they felt were similar to them through the Internet, sharing life events (Alianmoghaddam et al., 2019; Sundstrom, 2016) and contributing with their peer expertise (Wagg et al., 2022). This support led to less loneliness, embarrasment, and shame (Alianmoghaddam et al., 2019; Bäckström et al., 2021; Bernhardt & Felter, 2004; Clapton-Caputo et al., 2021; Johnson, 2015; Moon et al., 2019; Lupton, 2016), and increased learning and support (Bäckström et al., 2021; Bernhardt & Felter, 2004; Clapton-Caputo et al., 2021; Guerra-Reyes et al., 2016; Neill et al., 2014; Sundstrom, 2016). According to Guerra-Reyes et al. (2016), the relations on the Internet helped parents feel normal and created connections with other people. Particularly, concerning uncomfortable, private, and sensitive topics, controversial opinions, or “dumb” questions, connecting with people on the Internet was preferred (Guerra-Reyes et al., 2016; Johnson, 2015; Lupton, 2016; Moon et al., 2019) making it possible to stay safe (Madge & O’Connor, 2006; Wagg et al., 2022). Moreover, being availible anytime (Neill et al., 2014) for immediate affirmation and support (Moon et al., 2019) was valued. The possibility for social and emotional connection and support provided by people outside their social network was also valued (Clapton-Caputo et al., 2021; Criss et al., 2015; Johnson, 2015; Lupton, 2016; Rathbone & Prescott, 2019; Sundstrom, 2016). Specifically, using the words of Madge & O’Connor (2006) and Clapton-Caputo et al. (2021), parents formed anonymous connections to people while avoiding beeing judged. Hence, the Internet represented a community of reciprocity (Clapton-Caputo et al., 2021; Wagg et al., 2022) providing tools for respectful communication and partnership with people who shared their secrets and never rejected their knowledge or experiences (Clapton-Caputo et al., 2021; Griauzde et al., 2020).

### A novel Conceptual Understanding of Parental Experiences

We developed a lines-of-argument synthesis from the third order interpretations of the included 22 studies. The synthesis represents the core of parental experiences concerning use of the Internet for health information purposes and reads as follows: Parents experience the Internet as “A cyber partner for child caring” being a 24/7 available “go-to” among other confined sources. Parents find ways of “patching together” trustworthy information in solicitude for their child’s health while navigating between trust and anxiety. They relate online and share their experiences and secrets without being rejected.

## Discussion

The aim of this meta-ethnography of 22 studies was to synthesize the literature on how the parents of children aged below 5 years experienced using the Internet for health information purposes. The lines-of-argument synthesis point on how parents experience the Internet as “a cyber partner for child caring.” The metaphor of *“*partner” reflects several aspects of how the Internet is used as a companion and resource for health information in the exercising of the parental role, in developing health and information literacy coherent with parenting norms, and in safeguarding their children’s health. Moreover, the Internet as a “partner” intend to express how it is neither just a tool, nor a controlling device of others, but something parents, represented mainly by mothers, relate to independently and critically 24/7.

Mårtensson & Hensing’s (2012) approach to health literacy—that it can be complex and dynamic, and that it fluctuates—may further enlighten the current findings. Parents assess and make decisions regarding their children’s health on the Internet. Parents search the Internet in various ways, depending on their situational needs—sometimes they need information, sometimes they need support. Contrary to our preconceptions, we found that parents were concerned with finding reliable sources of health information on the Internet. They critically evaluated the information that they found, and tried to assess the websites that were familiar, reputable, and repeated, and that converged with other non-Internet sources in “patching together” what they timely needed. This adds knowledge to previous surveys that reported that parents do not critically evaluate the information they find to the fullest extent (Jaks et al., 2019; Yardi et al., 2018). Moreover, it flips the perspective from providing parents with proper information, into accepting their autonomy and competence, and rather acknowledge both parents and the Internet for their layperson and empowerment possibilities. As with other partners, it “takes two to tango” and the society and health services may provide both a better dance floor and music, metaphorically speaking. Throughout the two last decades of internet use and development, as well as reflecting contemporary parenting norms (Schmidt et al., 2023), we may expect experiences concerning the current topic of interest not to be easy or straightforward to understand or grasp. Our findings, and the synthesis across two decades of research, however, contributes with a deepened understanding of how connectivity, trust, weighing, and picturing, as suggested by Walker (2012), interact with information use and interpretations. While using the Internet for health information purposes as described in our findings, parents may also be able to stay true to the norms of being attentive and present, securing their child’s development and future, combining parenting and employment, being in control, and being contented/happy (Schmidt et al., 2023).

Even though the Internet was a resource many parents described positively, we also found that the Internet can make parents feel anxious, confused and conflicted, as with all important relationships and partners. This is consistent with the findings in the review by Kubb and Foran (2020). They also found that parents use internet to satisfy different needs for health information, and that it can be both beneficial, but also cause concern. In our work by writing this review, however, we have contributed to filling the gap about how and why parents search for health information on the Internet, and how it may empower parents to fulfill their role in relation to contemporary parenting norms. We also found that Internet can be used as support for parents, and that it is easily accessible and an effective way to gather health information as also described in the review concerning use of social media by Pretorius et al. (2019). In this meta-ethnography we also found that parents were motivated by the information they gathered on the internet, but it also created challenges for them, consistent with the review by Frey et al. (2022). The current meta-ethnography contributes to deepened understanding concerning use of the Internet including and beyond social media channels. Based on our study, we find no thorough argument of combating parental misinterpretation of information (based on an objective standard), as suggested by Frey et al. (2022). We do, however, acknowledge that trustworthy information, when needed, may be accessed and assessed by parents in collaboration with others including healthcare professionals, and that parents may be well equipped to develop both their information and health literacy as further discussed below.

Findings in the current study suggest that healthcare professionals need to meet parents on the Internet to communicate relevant and reliable health information, as parents will still turn to the Internet to assess such information anyway. Such initiatives have been taken, for instance as reported by Perkes et al. (2022), on their co-design with mothers, representing a disadvantaged population, of a safe and culturally adapted mobile health application, and Mobley et al. (2022) on their mobile health application addressing fathers to prevent obesity in their children. Our current findings also suggest that parents value and prefer validated sources, such as connecting with a healthcare professional on the Internet. Opportunities to assess trusted sources on the Internet could prevent parents from feeling confused, anxious and overwhelmed if the information is presented (by a trusted healthcare professional) in a usable, easily understandable and non-scientific language (Bernhardt & Felter, 2004).

The current findings may indicate that parents can use the Internet to educate themselves on parenting and child health-related issues. Parents aspire to being informed and want access to information that is tailored to their situation. The Internet enables them to access information and discuss health issues with other parents, and make well-founded decisions based on the knowledge they gained by searching the Internet, as also suggested by Mårtensson and Hensing (2012). By using the Internet for health information, not as a replace for other sources such as healthcare professionals, but as a supplement, they may be able to improve their parenting or to navigate as parents and “patch together” their own version of the information that is tailored to their own particular situation.

Although parents can have the capability and competence to navigate the Internet and to self-educate concerning health information, parents cannot be totally responsible for obtaining and understanding health information. The Internet should rather be viewed as an arena for sharing responsibility between parents and healthcare professionals, or metaphorically speaking, a dance floor where parents and healthcare professionals partner up. Rather than viewing the Internet as an obstacle for healthcare professionals, when it comes to cooperation with the parents on child health-related issues, it should instead be viewed as a platform to help improve parents’ feeling of empowerment and self-efficacy in the parenting role, so that they can become more health literate, and make healthy decisions and choices.

We found that parents used the Internet to seek social and emotional support, and shared experiences about parenthood and child health-related issues with other parents. This finding is also consistent with previous reviews and surveys (Dworkin et al., 2013; Lupton & Pedersen, 2016; Nicholl et al., 2017; Pretorius et al., 2019). The Internet is an environment in which parents never have to feel lonely, as they can always be in contact with other parents who are likeminded and in similar situations. The term “likeminded” and the experience it represents may though point in the direction of people who adjust and do not challenge, as well as people who interact on the premises of being in similar situations, but with different perspectives and experiences which they share for reciprocal enrichment. We would argue that both perspectives may be fruitful for parents, varying with situations and needs at time. Peer parents on the Internet were considered to be more honest and less restricted by guidelines, compared to healthcare professionals (Johnson, 2015). Our findings suggest that healthcare professionals cannot replace the experience of gathering around a shared life event, such as can be facilitated by the Internet. Peer parents’ stories and experiences were appreciated by parents and were partly an information resource for their own children’s health. We suggest that healthcare professionals should facilitate online resources for parents, where they can meet and discuss child health-related issues with other parents, and in which healthcare professionals participate by providing reliable health information.

### Strengths and Limitations

Only 34 of the total number of 650 participants in this study were fathers. Previous research has reported that women are more likely than men to seek health information on the Internet (Howard et al., 2001), and transferring the current findings to fathers should therefore be conducted with caution. Moreover, the 95% majority of mothers in the identified and included studies may reflect that fathers use the Internet less frequently to assess child health information as reported by Laws et al. (2019). There is a need of engaging fathers to a greater extent in research as well as childcaring as for instance done by Lee and Walsh (2015), by the use of Internet and technology innovation. In an analysis of father’s communication through social media, Kim et al. (2016) found that fathers as well obtain information on the Internet, discuss their concerns with peers, and receive emotional support. Fathers’ preferences and needs may differ though from mothers, relating to their gendered and family roles (Kim et al., 2016).

The dominance of highly educated participants in nine of the studies may possibly have dominated the findings on independent and critical use of the Internet reported. We examined the seven studies that included more participants with lower education levels and found that these studies did not alter the main findings from our meta-ethnographic synthesis. Through our preconceptions we might have been at risk of unintentionally magnifying confirming findings about frequent use of the Internet and difficulties in evaluating trustworthiness. We did, however, continuously verify our interpretations by the studies included. We made effort to stay grounded in the data and discussed this matter throughout the process to avoid interpretation biases by unconscious positioning.

Our review included studies from a wide variety of Western countries and India. The findings may hence be less transferable to most non-Western countries, as was the case also for previous reviews (Frey et al., 2022; Kubb & Foran, 2020; Pretorius et al., 2019). Another limitation in this study may be that it covers a wide timeframe (19 years) in which both parenting norms and advice from healthcare professionals may shift, and that the Internet is rapidly changing and evolving (Hillyer, 2020). Conversely, this could also be a strength, as our study included studies from 2004 to 2023, representing a wide range of Internet use and evolving parenting norms among parents throughout this period. Despite that experiences were reported in qualitative studies across a time span of 19 years, and heterogeneity concerning data collection and analysis methods, there was little variance between studies concerning experiences with using the Internet as a platform for health information purposes. This strengthens the meta-ethnography.

Although flaws identified by our quality assessment in 13 studies, the results of those studies aligned well with the nine studies without such flaws. Our overall interpretations and findings are hence supported by studies of good credibility which we suggest can be transferable for clinical recommendations as well as for a conceptual understanding among clinicians. We included 16 qualitative studies who were not included in previous reviews (Frey et al., 2022; Kubb & Foran, 2020; Pretorius et al., 2019), and our results both align with, and deepen previous knowledge. Another strength is that we applied recognized methods from Noblit and Hare (1988), and reported the steps transparently and verifiably, in accordance with the eMERGe reporting guidelines (France et al., 2019), which were developed collaboratively by experienced qualitative health researchers.

## Conclusion

The lines-of-argument synthesis represents a novel conceptual understanding and illustrates how parents experience using the Internet for health information purposes. Parents experience the Internet as “A cyber partner for child caring” being a 24/7 available “go-to” among other confined sources. Parents find ways of “patching together” trustworthy information in solicitude for their child’s health while navigating between trust and anxiety. They relate online and share their experiences and secrets without being rejected.

The Internet allows parents to “patch together” information in relation to experts, research, and peer parents’ experiences in order to fit their own needs by being tailored to their situation. The Internet enables them to obtain immediate information, social support and reassurance. The current findings and “partner” metaphor may flip the perspective from providing parents with current and accurate information into accepting their autonomy and competence, and rather acknowledge both parents and their peers on the Internet for their layperson and empowerment possibilities for “co-parenting.”

### Implications For Practice

The findings of this research may suggest that it is important for healthcare professionals to consider parents’ retrieval of health information from the Internet as an opportunity to enhance parental health literacy and access relevant information that can be tailored to complex, dynamic and fluctuating family and health situations. Parents request websites on the Internet and applications on their smartphones that enable them to access healthcare professionals 24/7 with their questions and concerns. Websites (or applications) can be ideal for healthcare professionals to communicate relevant, reliable, and tailored health information in a usable and easily understandable way, which might help to prevent parents from feeling overwhelmed and confused. Therefore, healthcare providers also need to have reputable lists of sources they can share with parents. Reliable websites that also include virtual communities with peer parents are also needed. The Internet could be viewed by healthcare professionals as a platform for parents to improve knowledge, feelings of empowerment and self-efficacy concerning their parenting role and access current child health information, which in turn may improve their children’s, as well as their own, health. The involvement of healthcare professionals on the Internet may also help to improve the services, as the Internet can facilitate familiarization with the parents’ problems, concerns, and interests.

### Implications For Research

Further research could incorporate fathers’ experiences of accessing and assessing child health information on the Internet, and similar research could also be conducted in non-Western countries and in all classes of society. The development and evaluation of science-based and reliable online health information resources, including virtual communities and access to healthcare staff, is warranted. Notably, based on the current meta-ethnography, a non-paternalistic approach to information and health literacy and parental use of the Internet could be maintained in future research. We acknowledge that the Internet may be used by parents both aligning to contemporary parenting norms, as well as groups feeling stigmatized and do not, while relating online. Examining the experiences of such groups, and how they relate on the Internet is also warranted.

## Supplemental Material

sj-docx-1-gqn-10.1177_23333936241259246 – Supplemental material for A meta-Ethnography on Parents’ Experiences of the Internet As a Source of Health Information Supplemental material, sj-docx-1-gqn-10.1177_23333936241259246 for A meta-Ethnography on Parents’ Experiences of the Internet As a Source of Health Information by Thale Strand and Thomas Westergren in Global Qualitative Nursing Research

sj-docx-2-gqn-10.1177_23333936241259246 – Supplemental material for A meta-Ethnography on Parents’ Experiences of the Internet As a Source of Health Information Supplemental material, sj-docx-2-gqn-10.1177_23333936241259246 for A meta-Ethnography on Parents’ Experiences of the Internet As a Source of Health Information by Thale Strand and Thomas Westergren in Global Qualitative Nursing Research

sj-docx-3-gqn-10.1177_23333936241259246 – Supplemental material for A meta-Ethnography on Parents’ Experiences of the Internet As a Source of Health Information Supplemental material, sj-docx-3-gqn-10.1177_23333936241259246 for A meta-Ethnography on Parents’ Experiences of the Internet As a Source of Health Information by Thale Strand and Thomas Westergren in Global Qualitative Nursing Research

sj-docx-4-gqn-10.1177_23333936241259246 – Supplemental material for A meta-Ethnography on Parents’ Experiences of the Internet As a Source of Health Information Supplemental material, sj-docx-4-gqn-10.1177_23333936241259246 for A meta-Ethnography on Parents’ Experiences of the Internet As a Source of Health Information by Thale Strand and Thomas Westergren in Global Qualitative Nursing Research

## References

[bibr1-23333936241259246] Abrahamsen M. H. Gramstad T. (2019). Google. In Store norske leksikon [Great Norwegian Encyclopedia]. http://snl.no/Google

[bibr2-23333936241259246] Alianmoghaddam N. Phibbs S. Benn C. (2019). “I did a lot of Googling”: A qualitative study of exclusive breastfeeding support through social media. Women and Birth, 32(2), 147–156. 10.1016/j.wombi.2018.05.008 29921552

[bibr3-23333936241259246] Altawil H. Klawunn R. Dierks M.-L. Lander J. (2023). Parental COVID-19-related health information practises, sources, evaluations and needs: A qualitative interview study. Health Expectations: An International Journal of Public Participation in Health Care and Health Policy, 26(1), 555–565. 10.1111/hex.13688 36482880 PMC9854324

[bibr4-23333936241259246] Aston M. Price S. Monaghan J. Sim M. Hunter A. Little V. (2018). Navigating and negotiating information and support: Experiences of first-time mothers. Journal of Clinical Nursing (John Wiley & Sons, Inc.), 27(3–4), 640–649. 10.1111/jocn.13970 28722771

[bibr5-23333936241259246] Atkins S. Lewin S. Smith H. Engel M. Fretheim A. Volmink J. (2008). Conducting a meta-ethnography of qualitative literature: Lessons learnt. BMC Medical Research Methodology, 8(1), 21. 10.1186/1471-2288-8-21 18416812 PMC2374791

[bibr6-23333936241259246] Bäckström C. Chamoun S. Tejani S. Larsson V. (2021). Parents’ perceptions about future digital parental support-A phenomenographic interview study. Frontiers in Digital Health, 3, 729697. 10.3389/fdgth.2021.729697 34778868 PMC8578718

[bibr7-23333936241259246] Bento A. I. Nguyen T. Wing C. Lozano-Rojas F. Ahn Y.-Y. Simon K. (2020). Evidence from internet search data shows information-seeking responses to news of local COVID-19 cases. Proceedings of the National Academy of Sciences of the United States of America, 117(21), 11220–11222. 10.1073/pnas.2005335117 32366658 PMC7260988

[bibr8-23333936241259246] Bernhardt J. M. Felter E. M. (2004). Online pediatric information seeking among mothers of young children: Results from a qualitative study using focus groups. Journal of Medical Internet Research, 6(1), e7. 10.2196/jmir.6.1.e7 PMC1550581 15111273

[bibr9-23333936241259246] Britten N. Campbell R. Pope C. Donovan J. Morgan M. Pill R. (2002). Using meta ethnography to synthesise qualitative research: A worked example. Journal of Health Services Research & Policy, 7(4), 209–215. 10.1258/135581902320432732 12425780

[bibr10-23333936241259246] Casilang C. G. Stonbraker S. Japa I. Halpern M. Messina L. Steenhoff A. P. Lowenthal E. D. Fleisher L. (2020). Perceptions and attitudes toward mobile health in development of an exclusive breastfeeding tool: Focus group study with caregivers and health promoters in the dominican republic. JMIR Pediatrics and Parenting, 3(2), e20312. 10.2196/20312 PMC7474414 32821063

[bibr11-23333936241259246] CASP (2018). Critical Appraisal Skills Programme. https://casp-uk.net/wp-content/uploads/2018/01/CASP-Qualitative-Checklist-2018.pdf

[bibr12-23333936241259246] Charmaz K. (2014). Constructing grounded theory (2nd ed.). Sage.

[bibr13-23333936241259246] Chu J. T. Wang M. P. Shen C. Viswanath K. Lam T. H. Chan S. S. C. (2017). How, when and why people seek health information online: Qualitative study in Hong Kong. Interactive Journal of Medical Research, 6(2), e7000. 10.2196/ijmr.7000 PMC5743920 29233802

[bibr14-23333936241259246] Clapton-Caputo E. Sweet L. Muller A. (2021). A qualitative study of expectations and experiences of women using a social media support group when exclusively expressing breastmilk to feed their infant. Women & Birth, 34(4), 370–380. 10.1016/j.wombi.2020.06.010 32674991

[bibr15-23333936241259246] Criss S. Woo Baidal J. A. Goldman R. E. Perkins M. Cunningham C. Taveras E. M. (2015). The role of health information sources in decision-making among Hispanic mothers during their children’s first 1000 days of life. Maternal and Child Health Journal, 19(11), 2536–2543. 10.1007/s10995-015-1774-2 26122256 PMC4596758

[bibr16-23333936241259246] Dworkin J. Connell J. Doty J. (2013). A literature review of parents’ online behavior. Cyberpsychology: Journal of Psychosocial Research on Cyberspace, 7(2), Article 2. 10.5817/CP2013-2-2

[bibr17-23333936241259246] Enli G. Aalen I. (2021). Facebook. In Store norske leksikon [Great Norwegian Encyclopedia]. http://snl.no/Facebook

[bibr18-23333936241259246] France E. F. Cunningham M. Ring N. Uny I. Duncan E. A. Jepson R. G. Maxwell M. Roberts R. J. Turley R. L. Booth A. Britten N. Flemming K. Gallagher I. Garside R. Hannes K. Lewin S. Noblit G. W. Pope C. Thomas J. Noyes J. (2019). Improving reporting of meta-ethnography: The eMERGe reporting guidance. Journal of Advanced Nursing, 75(5), 1126–1139. 10.1111/jan.13809 30644123 PMC7594209

[bibr19-23333936241259246] Frey E. Bonfiglioli C. Brunner M. Frawley J. (2022). Parents' use of social media as a health information source for their children: A scoping review. Academic Pediatrics, 22(4), 526−539. 10.1016/j.acap.2021.12.006 34906742

[bibr20-23333936241259246] Griauzde D. H. Kieffer E. C. Domoff S. E. Hess K. Feinstein S. Frank A. Pike D. Pesch M. H. (2020). The influence of social media on child feeding practices and beliefs among Hispanic mothers: A mixed methods study. Eating behaviors, 36, 101361. 10.1016/j.eatbeh.2019.101361 31923649 PMC8005295

[bibr21-23333936241259246] Guerra-Reyes L. Christie V. M. Prabhakar A. Harris A. L. Siek K. A. (2016). Postpartum health information seeking using mobile phones: Experiences of low-income mothers. Maternal and Child Health Journal, 20(S1), 13–21. 10.1007/s10995-016-2185-8 27639571 PMC5118389

[bibr22-23333936241259246] Henshaw E. J. Cooper M. A. Jaramillo M. Lamp J. M. Jones A. L. Wood T. L. (2018). “Trying to figure out if you’re doing things right, and where to get the info”: Parents recall information and support needed during the first 6 weeks postpartum. Maternal and Child Health Journal, 22(11), 1668–1675. 10.1007/s10995-018-2565-3 29978309

[bibr23-23333936241259246] Hillyer M. (2020). Here’s how technology has changed the world since 2000. World Economic Forum. https://www.weforum.org/agenda/2020/11/heres-how-technology-has-changed-and-changed-us-over-the-past-20-years/

[bibr24-23333936241259246] Howard P. E. N. Raine L. Jones S. (2001). Days and nights on the Internet: The impact of a diffusing technology. The American Behavioral Scientist, 45(3), 383–404. 10.1177/00027640121957259

[bibr25-23333936241259246] Jaks R. Baumann I. Juvalta S. Dratva J. (2019). Parental digital health information seeking behavior in Switzerland: A cross-sectional study. BMC Public Health, 19(1), 225. 10.1186/s12889-019-6524-8 30791927 PMC6385444

[bibr26-23333936241259246] Johnson S. A. (2015). ‘Intimate mothering publics’: Comparing face-to-face support groups and Internet use for women seeking information and advice in the transition to first-time motherhood. Culture, Health & Sexuality, 17(2), 237–251. 10.1080/13691058.2014.968807 25339096

[bibr27-23333936241259246] Kim H. N. Wyatt T. H. Li X. Gaylord M. (2016). Use of social media by fathers of premature infants. J Perinat Neonatal Nurs, 30(4), 359–366. 10.1097/jpn.0000000000000214 27776035

[bibr28-23333936241259246] Kubb C. Foran H. M. (2020). Online health information seeking by parents for their children: Systematic review and agenda for further research. Journal of Medical Internet Research, 22(8), e19985. 10.2196/19985 PMC7479585 32840484

[bibr29-23333936241259246] Laws R. Walsh A. D. Hesketh K. D. Downing K. L. Kuswara K. Campbell K. J. (2019). Differences between mothers and fathers of young children in their use of the internet to support healthy family lifestyle behaviors: Cross-sectional study. J Med Internet Res, 21(1), e11454. 10.2196/11454 PMC6364206 30674450

[bibr30-23333936241259246] Lee S. J. Walsh T. B. (2015). Using technology in social work practice: The mDad (Mobile Device Assisted Dad) case study. Advances in Social Work, 16(1), 107–124.

[bibr31-23333936241259246] Loignon C. Gottin T. Rahem N. Lafrenière D. Turcotte E. El Sherif R. Lagarde F. Doray G. Pluye P. (2022). Maternal experience with online information on parenting and infant care: Qualitative findings from Quebec, Canada. Journal of Child and Family Studies, 31(7), 1798–1808. 10.1007/s10826-021-02205-w

[bibr32-23333936241259246] Lupton D. (2016). The use and value of digital media for information about pregnancy and early motherhood: A focus group study. BMC Pregnancy and Childbirth, 16, 171. 10.1186/s12884-016-0971-3 27435182 PMC4950377

[bibr33-23333936241259246] Lupton D. Pedersen S. (2016). An Australian survey of women’s use of pregnancy and parenting apps. Women and Birth, 29(4), 368–375. 10.1016/j.wombi.2016.01.008 26874938

[bibr34-23333936241259246] Madge C. O’Connor H. (2006). Parenting gone wired: Empowerment of new mothers on the Internet? Social & Cultural Geography, 7(2), 199–220. 10.1080/14649360600600528

[bibr35-23333936241259246] Mårtensson L. Hensing G. (2012). Health literacy - a heterogeneous phenomenon: A literature review: Health literacy. Scandinavian Journal of Caring Sciences, 26(1), 151–160. 10.1111/j.1471-6712.2011.00900.x 21627673

[bibr36-23333936241259246] Maslen S. Harris A. (2021). Becoming a diagnostic agent: A collated ethnography of digital-sensory work in caregiving intra-actions. Social Science & Medicine, 277, 1–8. 10.1016/j.socscimed.2021.113927 33892417

[bibr37-23333936241259246] Mobley A. Gans K. Adamsons K. Zeldman J. Varela E. Whitlow L. (2022). O27 Pilot testing of a father-focused childhood obesity prevention mobile phone App. Journal of Nutrition Education and Behavior, 54(7, Supplement), S14. 10.1016/j.jneb.2022.04.034

[bibr38-23333936241259246] Moon R. Y. Mathews A. Oden R. Carlin R. (2019). Mothers’ perceptions of the Internet and social media as sources of parenting and health information: Qualitative study. Journal of Medical Internet Research, 21(7), e14289. 10.2196/14289 PMC6647756 31290403

[bibr39-23333936241259246] Negrone A. J. Caldwell P. H. Y. Scott K. M. (2023). COVID-19 and Dr. Google: Parents' changing experience using online health information about their children's health during the pandemic. Journal of Paediatrics and Child Health, 59(3), 512–518. https://doi.org/https://doi.org/10.1111/jpc.16339 36715457 10.1111/jpc.16339

[bibr40-23333936241259246] Neill S. J. Jones C. H. D. Lakhanpaul M. Roland D. T. Thompson M. J. , & the ASK SNIFF research team (2014). Parent’s information seeking in acute childhood illness: What helps and what hinders decision making? Health Expectations, 18(6), 3044–3056. 10.1111/hex.12289 25327454 PMC5810715

[bibr41-23333936241259246] Nicholl H. Tracey C. Begley T. King C. Lynch A. M. (2017). Internet use by parents of children with rare conditions: Findings from a study on parents’ web information needs. Journal of Medical Internet Research, 19(2), e5834. 10.2196/jmir.5834 PMC5350458 28246072

[bibr42-23333936241259246] Noblit G. W. Hare R. D. (1988). Meta-ethnography: Synthesizing qualitative studies. Sage.

[bibr43-23333936241259246] Page M. J. McKenzie J. E. Bossuyt P. M. Boutron I. Hoffmann T. C. Mulrow C. D. Shamseer L. Tetzlaff J. M. Akl E. A. Brennan S. E. Chou R. Glanville J. Grimshaw J. M. Hróbjartsson A. Lalu M. M. Li T. Loder E. W. Mayo-Wilson E. McDonald S. Moher D. (2021). The PRISMA 2020 statement: An updated guideline for reporting systematic reviews. BMJ, 372, n71. 10.1136/bmj.n71 PMC8005924 33782057

[bibr44-23333936241259246] Perkes S. J. Huntriss B. Skinner N. Leece B. Dobson R. Mattes J. Hall K. Bonevski B. (2022). Development of a maternal and child mHealth intervention with aboriginal and torres strait islander mothers: Co-design approach. JMIR Form Res, 6(7), e33541. 10.2196/33541 PMC9308065 35802404

[bibr45-23333936241259246] Pretorius K. Johnson K. E. Rew L. (2019). An integrative review: Understanding parental use of social media to influence infant and child health. Maternal and Child Health Journal, 23(10), 1360–1370. http://doi.org.ezproxy.uis.no/10.1007/s10995-019-02781-w 31222601 10.1007/s10995-019-02781-w

[bibr46-23333936241259246] Rathbone A. Prescott J. (2019). “I Feel Like A Neurotic Mother at Times”-a mixed methods study exploring online health information seeking behaviour in new parents. JMIR mHealth, 5, 14. 10.21037/mhealth.2019.05.02 PMC6624345 31380406

[bibr47-23333936241259246] Ritchie H. Mathieu E. Roser M. Ortiz-Ospina E. (2023). Internet. Retrieved 24.01.2024, from https://ourworldindata.org/internet

[bibr48-23333936241259246] Sharma N. Basu S. Manna S. Sharma P. Rao S. Duggal K. Kaur H. Kumar P. Malik S. T. (2022). Health-seeking behaviour for childhood ailments in caregivers of under-five children in an urban resettlement colony in Delhi, India. Cureus, 14(4), e24404. 10.7759/cureus.24404 PMC9126473 35619839

[bibr49-23333936241259246] Schmidt E.-M. Décieux F. Zartler U. Schnor C. (2023). What makes a good mother? Two decades of research reflecting social norms of motherhood. Journal of Family Theory & Review, 15(1), 57–77. https://doi.org/https://doi.org/10.1111/jftr.12488 38504801 10.1111/jftr.12488 PMC10947397

[bibr50-23333936241259246] Spinelli M. Lionetti F. Pastore M. Fasolo M. (2020). Parents' Stress and Children's Psychological Problems in Families Facing the COVID-19 Outbreak in Italy [Original Research]. Frontiers in Psychology, 11, 1713. 10.3389/fpsyg.2020.01713 32719646 PMC7350926

[bibr51-23333936241259246] Støren I. (2013). Bare søk! [Just search!] (2nd ed.). Cappelen Damm AS.

[bibr52-23333936241259246] Sundstrom B. (2016). Mothers “Google it Up”: Extending communication channel behavior in diffusion of innovations theory. Health Communication, 31(1), 91–101. 10.1080/10410236.2014.936339 26075413

[bibr53-23333936241259246] Tian X. Zhang S. (2022). Expert or experiential knowledge? How knowledge informs situated action in childcare practices. Social Science & Medicine (1982), 307, 115195. 10.1016/j.socscimed.2022.115195 35810691

[bibr54-23333936241259246] Tschamper M. K. Larsen M. H. Wahl A. K. Jakobsen R. (2023). Developing and maintaining health literacy: A continuous emotional, cognitive, and social process for parents of children with epilepsy—A qualitative study. Epilepsy & Behavior, 142, 109222. https://doi.org/https://doi.org/10.1016/j.yebeh.2023.109222 37088063 10.1016/j.yebeh.2023.109222

[bibr55-23333936241259246] van der Gugten A. C. de Leeuw R. J. R. J. Verheij T. J. M. van der Ent C. K. Kars M. C . (2016). E-health and health care behaviour of parents of young children: A qualitative study. Scandinavian Journal of Primary Health Care, 34(2), 135–142. 10.3109/02813432.2016.1160627 27063729 PMC4977935

[bibr56-23333936241259246] Wagg A. J. Hassett A. Callanan M. M. (2022). Exploring Online Social Support Groups, Part 2: “There's Just Pictures on Their Everyday and That's the Only Thing That Normalizes It for Me.” Clinical Lactation, 13(1), 24–31. 10.1891/CL.2021-0014

[bibr57-23333936241259246] Walker C. (2012). The Information World of Parents: A Study of the Use and Understanding of Information by Parents of Young Children. Library Trends, 60(3), 546-568. 10.1353/lib.2012.0000

[bibr58-23333936241259246] WHO (2021). Constitution of the world health organization. Retrieved October 18, 2021, from https://www.who.int/about/governance/constitution

[bibr59-23333936241259246] Yardi S. Caldwell P. H. Barnes E. H. Scott K. M. (2018). Determining parents’ patterns of behaviour when searching for online information on their child’s health. Journal of Paediatrics and Child Health, 54(11), 1246–1254. 10.1111/jpc.14068 29864197

[bibr60-23333936241259246] Yigzaw K. Y. Wynn R. Marco-Ruiz L. Budrionis A. Oyeyemi S. O. Fagerlund A. J. Bellika J. G. (2020). The association between health information seeking on the internet and physician visits (the seventh Tromsø study – part 4): Population-based questionnaire study. Journal of Medical Internet Research, 22(3), e13120. 10.2196/13120 PMC7082732 32134387

