# Supplementary material for: A meta-Ethnography on Parents’ Experiences of the Internet As a Source of Health Information
Source: Glob Qual Nurs Res. 2024 Jul 30;11:23333936241259246. doi: 10.1177/23333936241259246 (PMC11287733; doi:10.1177/23333936241259246)
Supplement: sj-docx-1-gqn-10.1177_23333936241259246 – Supplemental material for A meta-Ethnography on Parents’ Experiences of the Internet As a Source of Health Information [file sj-docx-1-gqn-10.1177_23333936241259246.docx]

**List of excluded full─texts with the following reason: Not possible to isolate findings from parents of children ≥6 years.**

Benedicta, B., Caldwell, P. H., & Scott, K. M. (2020). How parents use, search for and appraise online health information on their child's medical condition: A pilot study. *Journal of paediatrics and child health*, *56*(2), 252─258. https://doi.org/10.1111/jpc.14575

Bonanno, R., & Veselak, K. (2019). A Matter of Trust: Parents' Attitudes Toward Child Mental Health Information Sources [Article]. *Advances in Social Work*, *19*(2), 397─415. https://doi.org/10.18060/22970

Croucher, L., Mertan, E., Shafran, R., & Bennett, S. D. (2020). The Use of Mumsnet by Parents of Young People With Mental Health Needs: Qualitative Investigation. *JMIR mental health*, *7*(9), e18271. https://doi.org/10.2196/18271

Deuitch, N. T., Beckman, E., Halley, M. C., Young, J. L., Reuter, C. M., Kohler, J., Bernstein, J. A., Wheeler, M. T., Ormond, K. E., & Tabor, H. K. (2021). "Doctors can read about it, they can know about it, but they've never lived with it": How parents use social media throughout the diagnostic odyssey. *Journal of Genetic Counseling*, *30*(6), 1707─1718. https://doi.org/10.1002/jgc4.1438

Loignon, C., Gottin, T., Rahem, N., Lafrenière, D., Turcotte, E., El Sherif, R., Lagarde, F., Doray, G., & Pluye, P. (2022). Maternal Experience with Online Information on Parenting and Infant Care: Qualitative Findings from Quebec, Canada. *Journal of Child & Family Studies*, *31*(7), 1798─1808. https://doi.org/10.1007/s10826─021─02205─w

Malone, M., Mathes, L., Dooley, J., & While, A. E. (2005). Health information seeking and its effect on the doctor─patient digital divide. *Journal of telemedicine and telecare*, *11 Suppl 1*, 25─28. https://doi.org/10.1258/1357633054461831

Malone, M., While, A., & Roberts, J. (2014). Parental health information seeking and re─exploration of the 'digital divide'. *Primary health care research & development*, *15*(2), 202─212. https://doi.org/10.1017/S1463423613000194

Mason, A., Salami, B., Salma, J., Yohani, S., Amin, M., Okeke─Ihejirika, P., & Ladha, T. (2021). Health Information Seeking Among Immigrant Families in Western Canada. *Journal of pediatric nursing*, *58*, 9─14. https://doi.org/10.1016/j.pedn.2020.11.009

Negrone, A. J., Caldwell, P. H., & Scott, K. M. (2023). COVID─19 and Dr. Google: Parents' changing experience using online health information about their children's health during the pandemic. *Journal of paediatrics and child health*, *59*(3), 512─518. https://doi.org/10.1111/jpc.16339

Thorpe, M., Taylor, J., & Cole, R. (2021). Parents' use of information accessed through social media to make immunisation decisions for their young children. *Health promotion journal of Australia : official journal of Australian Association of Health Promotion Professionals*, *32*(2), 189─196. https://doi.org/10.1002/hpja.336

Tian, X., & Zhang, S. (2022). Expert or experiential knowledge? How knowledge informs situated action in childcare practices. *Social science & medicine (1982)*, *307*, 115195. https://doi.org/10.1016/j.socscimed.2022.115195
