# Supplementary material for: A meta-Ethnography on Parents’ Experiences of the Internet As a Source of Health Information
Source: Glob Qual Nurs Res. 2024 Jul 30;11:23333936241259246. doi: 10.1177/23333936241259246 (PMC11287733; doi:10.1177/23333936241259246)
Supplement: sj-docx-2-gqn-10.1177_23333936241259246 – Supplemental material for A meta-Ethnography on Parents’ Experiences of the Internet As a Source of Health Information [file sj-docx-2-gqn-10.1177_23333936241259246.docx]

**Table 1x.** Characteristics of the included studies

|  | **Sample**  (mothers/fathers) | **Age** **range** | **Higher** **educational** **level** **(%)** | **Data** **collection** | **Analysis** | **Phenomenon** **of** **interest** | **Explanation/** **Theory** |
| --- | --- | --- | --- | --- | --- | --- | --- |
| **Alianmoghaddam *et al.* (2019), New Zealand.** | 30/- | <25 − >35 | 80% | Interview | Aronson’s four stage thematic analysis methods | Inﬂuence of social media on breastfeeding practice | «Social constructionism» and the strength of «weak ties» |
| **Altawil *et al.* (2023), Germany.** | 22/8 | 18 − 50 | 40% | Interview | Qualitative content analysis | Parents search for COVID-19 (child)-specific health information and how they use and evaluate the information | Information seeking |
| **Aston *et al.* (2017), Canada.** | FG: 19 /-  EI: 18 / - | FG: 28/ EI: 31 (mean) | Not reported | Focus groups & E-interviews | Discourse analysis in accordance with feminist poststructuralism methodology | First-time mothers’ postpartum needs and where they went for information and support | Feminist poststructuralism |
| **Bäckström *et al.* (2021), Sweden.** | 11/4 | 23 − 57 years | 60% | Interview | Phenomenographic analysis | Parents’ perceptions of future digital parental support | Parental digital health literacy |
| **Bernhardt** **&** **Felter** **(2004),** **USA.** | 20/- | 22 − 42 | Not reported | Focus groups | Inductively using a "cut and paste" technique and deductively using relevant constructs from McGuire's Input/Output Matrix | Online health information and how to determine trust- worthiness | Information seeking |
| **Casilang *et al.* (2020), USA.** | 23/ - | 12 – 25: 15  26 – 54: 7  No answer: 1 | 17% | Focus groups & Interview | Deductive-inductive content analysis following theoretical framework of extended technology acceptance model and information-motivation-behavioral skills model | Use of information and communication technology, and attitudes and perceptions related to using mHealth | mHealth interventions |
| **Clapton-Caputo *et al.* (2021), Australia** | 10 / - | Not reported | Not reported | Interview | Thematic analysis by Braun & Clarke | Expectations and experiences of women who access social media groups when exclusively expressing breastmilk | Theory of Planned Behaviour |
| **Criss** ***et*** ***al.*** **(2015),** **USA.** | 49/- | Mean age 26,4 | 12% | Focus groups | Health Information Acquisition Model  Immersion/crystallization process | How health information sources inform decision- making | Socioeconomic status and race/ethnicity |
| **Griauzde *et al.* (2020), USA.** | 19 / - | 20 − 40+ | 26 % | Mixed method;  Interview | Directed content analysis (qualitative data) | Child feeding practices among Hispanic mothers and social media's role in that context | Socioeconomic status |
| **Guerra-** **Reyes** ***et*** ***al.*** **(2016),** **USA.** | 10/- | Mean age 29,9 | 80% | Interview | Content Analysis | Postpartum information needs | Health education |
| **van** **der** **Gugten** **e*t*** ***al.*** **(2016),** **Netherlands.** | 9/1 | Mean age 31,7 | 50% | Interview | Constant Comparison method. Structed in line with the process described in the Qualitative Analysis Guide of Leuven (QUAGOL) | Healthcare information on the Internet and its effect in decision making | Information seeking |

*Note. **Higher educational level = University or college degree, Abbreviations: *FG = Focus group. EI = E-Interview. mHealth = Mobile Health.*

**Table 1x (continued).** Characteristics of the included studies

|  | **Sample**  (mothers/fathers) | **Age** **range** | **Higher** **educational** **level** **(%)** | **Data** **collection** | **Analysis** | **Phenomenon** **of** **interest** | **Explanation/** **Theory** |
| --- | --- | --- | --- | --- | --- | --- | --- |
| **Henshaw** ***et*** ***al.*** **(2018),** **USA.** | 26/6  (1 sister) | Not reported | 89% | Focus groups | Constant Comparison Iterative Thematic Analysis | Adjusting to the parenting role, support, and information needs | Support and education needs |
| **Johnson** **(2015),** **Australia**. | 12/- | 29−44 | Most were university educated | Interview | Not reported | Support and information- seeking in ﬁrst-time motherhood | Virtual publics and counter- publics. A Feministic view |
| **Lupton** **(2016),** **Australia.** | 36/- | 23 − >40 | 72% | Focus groups | Inductiv thematic analysis | Digital media as a source of information about motherhood | Sociocultural and political aspects of digital media |
| **Madge** **&** **O’Connor,** **(2006),** **UK.** | Mothers (numbers not reported) | 22−36 | Not reported | Virtual group interviews | Not reported | Role of the Internet for mothers | Empowerment. A feministic view |
| **Maslen & Harris (2021), Australia.** | 25/12 | Not reported | 73 % | Fieldwork and Interview | A combination of thematic analysis and the generation of vignettes for a selection of participants | Digital-sensory work in contemporary healthcare | Karen Barad’s concept of ‘intra-action’ |
| **Moon,** ***et*** ***al.*** **(2019),** **USA.** | 28/- | 20−44 | 75% | Focus groups and individual interviews | Standard qualitative analytic techniques and a grounded theory approach with line-by-line coding | Digital media as sources of parenting and health information | Infant care decisions making |
| **Neill** ***et*** ***al.*** **(2014),** **UK.** | 24/3 | <30− 49 | 63% | Focus groups and individual interviews | Constant comperative analysis | Information resources during decision making | Decision making |
| **Rathbone & Prescott (2019), UK.** | 153/- | 26─ >41 | Not reported | Mixed method; Open ended questionnaire | Thematic analysis by Braun & Clarke | Why new parents turn to the online platform for health information | Health anxiety |
| **Sharma *et al.* (2022), India.** | 17 / - | Not reported | 47% | Interview | Thematic analysis utilizing the Theory of planned behavior | The health-seeking behavior for childhood aliments in caregivers | Theory of Planned Behaviour |
| **Sundstrom** **(2016),** **USA.** | 44/- | 18−40 | 43% | Interview | Grounded theory approach including the Diffusions of innovations theory, and line-by-line coding | Use of information resources in relation to health problems | «Diffusion of Innovations» theory and “Weak ties” |
| **Wagg *et al.* (2022), UK.** | 10 / - | Not reported | Not reported | Interview | Interpretative Phenomenological Analysis | Experiences of using online social support via Facebook, capture the meaning that they assign to the groups, and the perceived impact that the groups have on breastfeeding decisions and their journey | Emotional, technical, informational, and experiential support |

*Note. **Higher educational level = University or college degree, Abbreviations: *FG = Focus group. EI = E-Interview. mHealth = Mobile Health.*
