# Supplementary material for: A meta-Ethnography on Parents’ Experiences of the Internet As a Source of Health Information
Source: Glob Qual Nurs Res. 2024 Jul 30;11:23333936241259246. doi: 10.1177/23333936241259246 (PMC11287733; doi:10.1177/23333936241259246)
Supplement: sj-docx-3-gqn-10.1177_23333936241259246 – Supplemental material for A meta-Ethnography on Parents’ Experiences of the Internet As a Source of Health Information [file sj-docx-3-gqn-10.1177_23333936241259246.docx]

**Table 4.** Concept: The 24/7 available ‘go-to’ among other confined sources

|  | **THE 24/7 AVAILABLE ‘GO-TO’ AMONG OTHER CONFINED SOURCES** | |
| --- | --- | --- |
|  | *Unlimited, accessible, and additional* | *Parental preferences for the ‘go-to’* |
| **Alianmoghaddam *et al.* (2019). New Zealand.** | Practical and convenient. Affordable and timely accessible without professional availability. Trusting health professionals | Smartphone screens, apps, and social media |
| **Altawil *et al.* (2023). Germany.** | Helpful, accurate, detailed, easily accessible and sufficient information. Using to look for more detailed information. Trusting pediatricians and family physicians. Others opinions influenced their own opinion and information decisions | A more transparent and up-to-date information and also a direct, personal interaction with health personnel online |
| **Aston *et al.* (2017). Canada.** | Finding information from different resources. Evaluating and comparing different sources of information to make decisions | - |
| **Bäckström *et al.* (2021). Sweden.** | Easily accessible information that is comprehensive and free of charge | Opportunities for parents to interact and communicate with health professionals online to receive reliable answers immediately |
| **Bernhardt** **&** **Felter** **(2004).** **USA.** | Getting advice when the doctor’s office is not reachable and to clarify or supplement information received from the doctor. Trusting health professionals and other parents. Deciding if they need to seek help or not | Web-based tailored messages |
| **Casilang *et al.* (2020). USA.** | Confirming received information, finding further research, and finding information. Trusting healthcare professionals. Helpful in decision-making | Easily accessible information about child health with an interactive component and simple, educational, and motivational messaging with video and images |
| **Clapton-Caputo *et al.* (2021). Australia** | The ability to access emotional support 24/7 depending on their own needs. Trusting other mothers. Getting practical information and multiple opinions to use to guide their own decisions | - |
| **Criss** ***et*** ***al.*** **(2015).** **USA.** | Immediate information when a healthcare provider is not available. Reviewed various sources to confirm information and address discrepancies. Trusting health professionals | Links to informative websites, others’ experiences, and ability to ask questions |
| **Griauzde *et al.* (2020).USA.** | Using social media and applications for information | - |
| **Guerra-** **Reyes** ***et*** ***al.*** **(2016).** **USA.** | Readily, accessible, and faster source of information than medical professionals. Using multiple Information steams. Trusting expert advice and people they trusted | Lacking available apps with postpartum content or awareness |
| **van** **der** **Gugten** **e*t*** ***al.*** **(2016).** **Netherlands.** | Easily accessible 24 h a day. A complement to what they had been told at the physician. Trusting physicians face-to-face or medical institutions or organizations online. Could postpone a doctor's visit. Decision based on own judgment. | Tailor-made information |
| **Henshaw** ***et*** ***al.*** **(2018).** **USA.** | - | An interactive, tailored online or telehealth program |
| **Johnson** **(2015).** **Australia**. | Searching when needing more information than provided from medical professionals. Trusting practical advice based on lived experience. Seeking advice they normally go to a doctor for to get decision support | - |
| **Lupton** **(2016).** **Australia.** | Immediate, quick, reassuring, repeatable, and timesaving without disturbing others and without engaging. Trusting other parents and online sources with government backing | Apps, wearable tracking devices and question-and-answers with health professionals available 24/7. Desired greater access to advice and support from health professionals |
| **Madge** **&** **O’Connor** **(2006).** **UK.** | Immediate information and support at unsociable hours. A supplement to information gained from books and health professionals | - |
| **Maslen & Harris (2021). Australia.** | Searching online following clinical encounter to re-think | - |
| **Moon** ***et*** ***al.*** **(2019).** **USA.** | Unlimited amount of information without bothering others. Confirming information obtained from other sources. Trusting forums and social media. Multiple opinions guiding their decision | Tailored information |
| **Neill** ***et*** ***al.*** **(2014).** **UK.** | Supplement information gathered at the doctor's office. Searching when having an actual diagnosis | Ease of access and professional validation of the content |
| **Rathbone & Prescott (2019). UK.** | Online searching for plethora of aspects | - |
| **Sharma *et al.* (2022). India.** | The internet is used among other information sources. Trusting healthcare professionals. | - |
| **Sundstrom** **(2016).** **USA.** | Using multiple Communications Channels. Trusting layperson expertise | - |
| **Wagg *et al.* (2022). UK.** | Online support is easier to access | - |

**Table 5.** Concept: ‘Patching together’ trustworthy information with solicitude and concern

|  | **‘PATCHING TOGETHER’ TRUSTWORTHY INFORMATION WITH SOLICITUDE AND CONCERN** | |
| --- | --- | --- |
|  | *Critically ‘patching together’* | *Doubt, anxiousness, and inner conflict* |
| **Alianmoghaddam *et al.* (2019). New Zealand.** | Improving own health knowledge and self-confidence | Doubts about information reliability |
| **Altawil *et al.* (2023). Germany.** | Deciding which information to use | Tried to determine trustworthiness. Difficult to identify the reliability of online sources because of its multiple conflicting opinions. Uncertain, confusing, false, and contradictory information |
| **Aston *et al.* (2017). Canada.** | Critically analyzing the information to help make well-informed decisions that resonated with their individual beliefs, values, and practices | Selective of what information they did or did not accept |
| **Bäckström *et al.* (2021). Sweden.** | - | - |
| **Bernhardt** **&** **Felter** **(2004).** **USA.** | Learning more. Informed treatment decisions | Difficult to evaluate reliability. Trustworthiness increased with familiar and repeated sources. Could cause concerns |
| **Casilang *et al.* (2020). USA.** | Information validated by credible sources | - |
| **Clapton-Caputo *et al.* (2021). Australia.** | Reliable and credible information from other mothers. Getting information through experiences that they felt that healthcare professionals did not have the correct information about | - |
| **Criss** ***et*** ***al.*** **(2015).** **USA.** | Used multiple sources for consistency and resolving contradictory information. Trusted info that converged with non-internet sources | - |
| **Griauzde *et al.* (2020). USA.** |  | Avoiding social media and internet because it is not trustworthy |
| **Guerra-** **Reyes** ***et*** ***al.*** **(2016).** **USA.** | Educational level affected where they found information and what they used the information for | Apps were repetitive, non-validated, or contained irrelevant content |
| **van** **der** **Gugten** **e*t*** ***al.*** **(2016).** **Netherlands.** | Developed a frame of reference and increased understanding. Removing insecurity, confirming own ideas, check on information and an extra opinion. Trusted repeated sources and tried to seek out the background | Doubts about the reliability. Enormous amount of information that could evoke insecurities, doubts, and anxiousness |
| **Henshaw** ***et*** ***al.*** **(2018).** **USA.** | - | Challenging finding reliable, non-judgmental info and deciding what to trust. Too much info that could lead to feeling conflicted, confused, or concerned |
| **Johnson** **(2015).** **Australia**. | Patch together their own version of motherhood | The wealth of information overwhelming and confusing |
| **Lupton** **(2016).** **Australia.** | Valued customized, personalized and detailed information. | Avoided sources that could create anxiety |
| **Madge** **&** **O’Connor** **(2006).** **UK.** | Increased confidence and greater sense of control leading to feeling of empowerment |  |
| **Maslen & Harris (2021). Australia.** | - | - |
| **Moon** ***et*** ***al.*** **(2019).** **USA.** | Quickly crowdsource or gather multiple viewpoints. Convenient place to obtain information that was not urgent. Information on websites as generally trustworthy | Overwhelming amount of information |
| **Neill** ***et*** ***al.*** **(2014).** **UK.** | Judged the quality when searching for information on the internet | Conflicting info that could lead to feeling uncertain and anxious |
| **Rathbone & Prescott (2019). UK.** | Searching in self-doubt | - |
| **Sharma *et al.* (2022). India.** | Tendency of self-managing | Information on the internet may lack credibility and authenticity |
| **Sundstrom** **(2016).** **USA.** | Evaluating to find reliable and trustworthy sources | - |
| **Wagg *et al.* (2022). UK.** | Massive reassurance from peers' experiences. Reassurance in groups to oppose professionals when needed | - |

**Table 6.** Concept: Relating online through shared experiences

|  | **RELATING ONLINE THROUGH SHARED EXPERIENCES** |
| --- | --- |
| **Alianmoghaddam *et al.* (2019). New Zealand.** | Come together on Facebook around a shared life event. Efficient source of social support and encouragement. Prevented feeling of social isolation. Advising and supporting others |
| **Altawil *et al.* (2023). Germany.** | - |
| **Aston *et al.* (2017). Canada.** | - |
| **Bäckström *et al.* (2021). Sweden.** | Learning from other parents. Supporting each other. Preventing feeling of loneliness |
| **Bernhardt** **&** **Felter** **(2004).** **USA.** | Social support. Appreciated sharing stories so that they did not feel alone |
| **Casilang *et al.* (2020). USA.** | - |
| **Clapton-Caputo *et al.* (2021). Australia.** | Shared experiences reduced their self-blame and feeling of isolation. Emotional support. Non-judgmental. Connecting on an emotional level. Reciprocal support and respective tone |
| **Criss** ***et*** ***al.*** **(2015).** **USA.** | Social networking on FB, but not discussing health information. Info from outside their social network |
| **Griauzde *et al.* (2020).USA.** | SoMe and text messaging as tools for communication |
| **Guerra-** **Reyes** ***et*** ***al.*** **(2016).** **USA.** | Feeling of normality and connection to other people. Reassurance and social support on FB. Difficult or uncomfortable topics |
| **van** **der** **Gugten** **e*t*** ***al.*** **(2016).** **Netherlands.** | - |
| **Henshaw** ***et*** ***al.*** **(2018).** **USA.** | - |
| **Johnson** **(2015).** **Australia**. | Valued people’s thoughts and experiences. Developing intimacy. Support when having controversial opinions. Safe space and avoiding embarrassment |
| **Lupton** **(2016).** **Australia.** | Provided intimacy. Search for specialist groups on FB. Share personal details and experiences, developing relationships and dealing with isolation. Share private or sensitive topics |
| **Madge** **&** **O’Connor** **(2006).** **UK.** | Feeling of ‘shared experience’. A safe space to try out their new identity as a mom. Empowerment. Forming connections. Anonymity and non- judgmental |
| **Maslen & Harris (2021). Australia.** | - |
| **Moon** ***et*** ***al.*** **(2019).** **USA.** | Not wanting to ask “dumb” or too personal questions. Immediate affirmation and support |
| **Neill** ***et*** ***al.*** **(2014).** **UK.** | Social support during antisocial hours |
| **Rathbone & Prescott (2019). UK.** | Normalization |
| **Sharma *et al.* (2022). India.** | - |
| **Sundstrom** **(2016).** **USA.** | Valued other people’s experiences. Social and emotional support |
| **Wagg *et al.* (2022). UK.** | Peer expertise and safe likeminded. Similar experiences. Appearing normal and avoid judgment. Reassurance in groups to oppose professionals when needed. Community of reciprocity |
